# A mental health survey among young front-line clinicians in high-risk areas during the COVID-19 sporadic epidemic in China

**DOI:** 10.3389/fpsyt.2022.872331

**Published:** 2022-08-30

**Authors:** Xingbo Suo, Yang Zhang, Qingxia Liu, Gaofeng Zhao, Yanan Zhu, Yan Liu, Jinguo Zhai

**Affiliations:** ^1^Department of Psychiatry, Jining Medical University, Jining, China; ^2^Department of Psychiatry, Harbin Medical University, Harbin, China; ^3^Shandong Daizhuang Hospital, Jining, China; ^4^Harbin First Hospital, Harbin, China

**Keywords:** COVID-19, young front-line clinicians, mental health, sporadic, high-risk areas

## Abstract

**Background:**

The sporadic coronavirus disease (COVID-19) epidemic has placed enormous psychological stress on people, especially clinicians. The objective of this study was to examine depression, anxiety, quality of life (QOL), and related social psychological factors among young front-line clinicians in high-risk areas during the COVID-19 sporadic epidemic in China and to provide a reference for formulating reasonable countermeasures.

**Methods:**

In this cross-sectional study, demographic information, COVID-19-related questions, anxiety (Generalized Anxiety Disorder-7, GAD-7), depression (Patient Health Questionnaire-9, PHQ-9), insomnia (Insomnia Severity Index, ISI), stress (Perceived Stress Scale-10, PSS-10), and QOL (World Health Organization Quality of Life-brief version, WHOQOL-BREF) were collected. Binary logistic regression analysis was used to test the relationships between anxiety and/or depression and other related problems. Multiple linear regression analysis was used to test the relationships among factors influencing QOL.

**Results:**

A total of 146 young front-line clinicians were included. The prevalence rates of depression, anxiety, and anxiety-depression comorbidity were 37.7% (95% CI = 29.7–45.6%), 26.0% (95% CI = 18.8–33.2%), and 24.0% (95% CI = 17.0–31.0%), respectively. Severe stress (OR = 1.258, 95% CI = 1.098–1.442, *P* < 0.01) and insomnia (OR = 1.282, 95% CI = 1.135–1.447, *P* < 0.01) were positively correlated with depression. Severe stress (OR = 1.487, 95% CI = 1.213–1.823, *P* < 0.01) and insomnia (OR = 1.131, 95% CI = 1.003–1.274, *P* < 0.05) were positively correlated with anxiety. Severe stress (OR = 1.532, 95% CI = 1.228–1.912, *P* < 0.01) was positively correlated with anxiety-depression comorbidity. However, insomnia (OR = 1.081, 95% CI = 0.963–1.214, *P* > 0.05) was not correlated with anxiety-depression comorbidity. The belief that the vaccine will stop the COVID-19 pandemic (OR = 0.099, 95% CI = 0.014–0.715, *P* < 0.05) was negatively correlated with anxiety and anxiety-depression comorbidity (OR = 0.101, 95% CI = 0.014–0.744, *P* < 0.05). Severe stress (B = −0.068, 95% CI = −0.129 to −0.007, *P* < 0.05) and insomnia (B = −0.127, 95% CI = −0.188 to −0.067, *P* < 0.01) were negatively correlated with QOL. The belief that the vaccine could provide protection (B = 1.442, 95% CI = 0.253–2.631, *P* < 0.05) was positively correlated with QOL.

**Conclusions:**

The prevalence of depression, anxiety, and even anxiety-depression comorbidity was high among young front-line clinicians in high-risk areas during the COVID-19 sporadic epidemic in China. Various biological and psychological factors as well as COVID-19-related factors were associated with mental health issues and QOL. Psychological intervention should evaluate these related factors and formulate measures for these high-risk groups.

## Introduction

In March 2020, the World Health Organization (WHO) announced coronavirus disease (COVID-19) to be a pandemic (1). Globally, to date (January 4, 2022), this destructive pandemic has spread rapidly across 226 countries/regions, and 296,496,809 confirmed cases of COVID-19 have been reported to the WHO (2). To contain this global outbreak, the Chinese government adopted a series of strict and effective public health measures, such as encouraging people to wear protective masks, self-isolation, and the cancellation of mass gatherings (3). At present, the epidemic situation in China has now largely been brought under control, and epidemic prevention and control have become the norm (4). However, there are still sporadic cases that occur in some places in China, and a higher risk of infection and stricter isolation measures were borne by the people in these areas. Due to the spread of the COVID-19 sporadic epidemic, parts of Harbin Municipality have been defined as high-risk areas of the epidemic since September 25, 2021. There was no doubt that it would seriously affect the local people's mental health and quality of life (3).

Clinicians are at the core of epidemic preparedness and control in high-risk areas during periods of sporadic epidemic situations. In contrast to the general population, front-line clinicians may have greater psychological stress in high-risk areas during the COVID-19 sporadic epidemic. Multiple past studies have demonstrated that during the Severe Acute Respiratory Syndrome (SARS), Influenza A (H1N1), and Middle East Respiratory Syndrome (MERS) outbreaks, front-line medical staff were at higher risk of psychological problems, including but not limited to anxiety, depression, and posttraumatic stress disorder (PTSD) (5–11). They are predisposed to high workloads, unpredictable work patterns, and a higher risk of infection (12–16). In addition, clinicians are easily ostracized by people around them after work because the general population easily misunderstands that clinicians are especially susceptible to carrying the virus when returning home (14). Clinicians may also be worried about becoming infected or infecting their families (14). Stress from these various sources will increase the risk for depression or anxiety if it cannot be effectively allayed (15, 17). Moreover, previous studies have shown that compared with older clinicians, young clinicians' lack of practical experience and confidence in clinical management may lead to insufficient resilience to deal with psychological problems and more serious emotional exhaustion (18, 19). Therefore, mental health conditions such as depression and anxiety may be worse among young front-line clinician populations than among senior clinician populations (20–22). In addition, the term “quality of life” (QOL) is the subjective perception of wellbeing and wholeness (23). Due to the lack of evidence-based practice related to sporadic epidemic management, even less is known about the factors that worsen or improve QOL. A study has shown that young people may have poorer QOL relative to older people during the COVID-19 epidemic (24). The mental health status and QOL of young front-line clinicians should receive more attention, so we chose this group as the main study population. Although many research articles on the psychological status and QOL of clinicians have been published during the epidemic (25–30), there has been no study on young front-line clinicians during the COVID-19 sporadic epidemic in high-risk areas.

Currently, there is a need for testimony of mental health problems during the sporadic epidemic situation to identify those at high risk and to investigate the related psychological factors and social resources that can alleviate this threat. Therefore, we carried out this study to examine depression, anxiety, QOL, and related social psychological factors among young front-line clinicians in high-risk areas during the COVID-19 sporadic epidemic in China.

## Methods

### Participants

Participants who met the following eligibility criteria were included: (1) clinicians, (2) aged between 18 and 40 years, (3) could read a Chinese questionnaire, and (4) WeChat users. Informed consent was obtained from all subjects before filling in the questionnaire, and this study was approved by the Research Ethics Committee of the Shandong Daizhuang Hospital (Second Affiliated Hospital of Jining Medical University) in Shandong China.

The sample size was calculated with the following formula (31): *N* = (Zα^2^ × *P* × (1–*P*))/d^2^. The confidence level (Z) was equal to 1.96 at the significance level of α = 0.05, P was the estimated proportion, and d was the tolerated margin of error and was calculated to be 0.10. A previous study found depression and anxiety prevalence rates to be 27.9 and 31.6%, respectively, in the general population (32). As no study has shown the prevalence of anxiety and depression among young Chinese clinicians during the COVID-19 pandemic, to achieve sufficient statistical power, we used *P* = 0.279 to calculate the sample size and found 77 subjects to be needed in this study.

### Data collection

This cross-sectional study was conducted between September 27th and 30th, 2021, in two hospitals in Harbin Municipality, Heilongjiang Province in China. Due to the risk of infection during the COVID-19 pandemic, face-to-face interviews could not be executed. In this study, we used an online-based survey *via* the WeChat-based survey program “Questionnaire Star” to collect data (33), mainly drawing on the fact that WeChat is the largest social communication media with more than 1 billion users in China (34). In the study, our research assistants forwarded the questionnaire to various WeChat groups of young clinicians to collect information. The questionnaire required each question be answered before it could be submitted. The same IP address could be used only once to complete the questionnaire.

### Measurements

#### Sociodemographic variables

Using the questionnaire, we collected sociodemographic data, including gender, marriage, education level, inhabitation, and fertility.

#### Explanatory variables

Following previous studies on the influenza vaccine (35, 36), several standardized questions related to COVID-19 were used in this study, including (1) “Do you worry about family and friends being infected with COVID-19?” (No/Fair/Very much); (2) “Do you think COVID-19 vaccines could protect you from COVID-19?” (No/No idea/Yes); (3) “What do you think of the long-term side effects of the COVID-19 vaccines?” (Not safe with obvious side effects/No idea/Safe with no or minimal side effects); and (4) “What do you think the vaccine will stop the global epidemic?” (No/No idea/Yes).

Insomnia severity was assessed by the validated Chinese version of the 7-item Insomnia Severity Index (ISI), which has been widely used in clinical research, with a total score ranging from 0 to 28. Insomnia was defined with a cutoff point of 8, i.e., ISI ≥ 8 (37, 38). The Cronbach's alpha of the scale was 0.842.

Stress severity was assessed by the validated Chinese version of the 10-item Perceived Stress Scale (PSS-10), which has been widely used in clinical research with a total score ranging from 0 to 40. Higher scores indicate greater stress severity (39, 40). The Cronbach's alpha of the scale was 0.915.

#### Outcome variables

Depression severity was assessed by the validated Chinese version of the 9-item Patient Health Questionnaire (PHQ-9), which has been widely used in clinical research, with a total score ranging from 0 to 27. Depression was defined with a cutoff point of 5, i.e., PHQ-9 ≥ 5 (37, 41). The Cronbach's alpha of the scale was 0.896.

Anxiety severity was assessed by the validated Chinese version of the 7-item Generalized Anxiety Disorder (GAD-7), which has been widely used in clinical research, with a total score ranging from 0 to 21. Anxiety was defined with a cutoff point of 5, i.e., GAD-7 ≥ 5 (42, 43). Anxiety-depression comorbidity was defined with a cutoff point of 5, i.e., both PHQ-9 ≥ 5 and GAD-7 ≥ 5. The Cronbach's alpha of the scale was 0.945.

The overall QOL was assessed by the sum of the first two item scores of the Chinese version of the World Health Organization Quality of Life-brief version (WHOQOL-BREF), with a total score ranging from 2 to 10. Higher scores indicate a greater QOL (44). The Cronbach's alpha of the scale was 0.801.

### Data analysis

All data were analyzed by using the Statistical Package for Social Science (SPSS) version 25.0. EXCEL was adopted to manage the data. Because the diseases of the subjects were different, we compared demographic variables and questionnaires between the anxiety-depression comorbidity and no anxiety or depression groups, between the anxiety and no anxiety groups, and between the depression and no depression groups. Categorical variables were compared by the chi-square test. Shapiro Wilk (S-W) was used to test the normality of quantitative variables. The variables that were compliant with normality were subjected to independent *t*-tests, while those that did not meet normality were subjected to Mann–Whitney U tests. Variables with statistical significance in the significance test were included in the binary logistic regression analysis, which was used to identify the factors associated with depression, anxiety, and anxiety-depression comorbidity. Spearman's rank-order analysis was used to test the relationship between depression and anxiety. Multiple linear regression analysis was used to assess the associations of factors influencing QOL. Statistical significance tests were two-tailed, and *P* < 0.05 was considered statistically significant.

## Results

### Sociodemographic and clinical characteristics

A total of 154 young front-line clinicians were enrolled in the current analysis. A total of 146 participants met the inclusion criteria and were finally included in our study, with a response rate of 94.8%. The sociodemographic and clinical characteristics of the study participants are shown in Table 1. Regarding sociodemographic characteristics, 61.64% (*n* = 90) of the total sample were female clinicians.

**Table 1:** The sociodemographic and clinical characteristics of the study participants.

**Variable**	**No DEP (*N* = 91) *N*%**	**DEP (*N* = 55) *N*%**	***P***	**No ANX (*N* = 108) *N*%**	**ANX (*N* = 38) *N*%**	***P***	**No DEP or ANX (*N* = 111) *N*%**	**Comorbid DEP and ANX (*N* = 35) *N*%**	***P***
Gender (female)	59 (64.8)	31 (56.4)	0.308	68 (63.0)	22 (57.9)	0.581	71 (64.0)	19 (54.3)	0.305
**Education level**									
Bachelor's degree	23 (25.3)	8 (14.5)	0.040^*^	25 (23.1)	6 (15.8)	0.352	26 (23.4)	5 (14.3)	0.132
Master's degree	57 (62.6)	45 (81.8)		72 (66.7)	30 (78.9)		73 (65.8)	29 (82.9)	
Doctoral degree	11 (12.1)	2 (3.6)		11 (10.2)	2 (5.3)		12 (10.8)	1 (2.9)	
Marriage (single)	77 (84.6)	49 (89.1)	0.446	91 (84.3)	35 (92.1)	0.226	94 (84.7)	32 (91.4)	0.312
Fertility (none)	83 (91.2)	53 (96.4)	0.232	99 (91.7)	37 (97.4)	0.231	102 (91.9)	34 (97.1)	0.284
**Inhabitation**									
Alone	17 (18.7)	9 (16.4)	0.001^**^	22 (20.4)	4 (10.5)	0.011^*^	22 (19.8)	4 (11.4)	0.003^**^
With family	33 (36.3)	6 (10.9)		34 (31.5)	5 (13.2)		36 (32.4)	3 (8.6)	
Others	41 (45.1)	40 (72.7)		52 (48.1)	29 (76.3)		53 (47.7)	28 (80.0)	
**Worried about being infected with COVID-19**									
No	33 (36.3)	14 (25.5)	0.149	39 (36.1)	8 (21.1)	0.019^*^	39 (35.1)	8 (22.9)	0.046^*^
Fair	49 (53.8)	30 (54.5)		59 (54.6)	20 (52.6)		61 (55.0)	18 (51.4)	
Very much	9 (9.9)	11 (20.0)		10 (9.3)	10 (26.3)		11 (9.9)	9 (25.7)	
**Thought COVID-19 vaccines could provide protection**									
No	20 (22.0)	14 (25.5)	0.403	26 (24.1)	8 (21.1)	0.232	26 (23.4)	8 (22.9)	0.303
No idea	65 (71.4)	40 (72.7)		75 (69.4)	30 (78.9)		78 (70.3)	27 (77.1)	
Yes	6 (6.6)	1 (1.8)		7 (6.5)	0 (0.0)		7 (6.3)	0 (0.0)	
**Though vaccines are safe**									
Not safe with obvious side effects	7 (7.7)	13 (23.6)	0.012^*^	11 (10.2)	9 (23.7)	0.018^*^	12 (10.8)	8 (22.9)	0.039^*^
No idea	69 (75.8)	38 (69.1)		79 (73.1)	28 (73.7)		81 (73.0)	26 (74.3)	
Safe with no or minimal side effects	15 (16.5)	4 (7.3)		18 (16.7)	1 (2.6)		18 (16.2)	1 (2.9)	
**Though vaccines will stop the global epidemic**									
No	14 (15.4)	16 (29.1)	0.101	19 (17.6)	11 (28.9)	0.040^*^	19 (17.1)	11 (31.4)	0.040^*^
No idea	58 (63.7)	32 (58.2)		65 (60.2)	25 (65.8)		68 (61.3)	22 (62.9)	
Yes	19 (20.9)	7 (12.7)		24 (22.2)	2 (5.3)		24 (21.6)	2 (5.7)	
	**M (Q)**	**M (Q)**	***P***	**M (Q)**	**M (Q)**	***P***	**M (Q)**	**M (Q)**	***P***
Insomnia	2.0 (5.0)	7.0 (6.0)	<0.001^***^	3.0 (5.0)	7.0 (8.0)	<0.001^***^	3.0 (5.0)	7.0 (6.0)	<0.001^***^
	**μ** **(SD)**	**μ** **(SD)**	***P***	**μ** **(SD)**	**μ** **(SD)**	***P***	**μ** **(SD)**	**μ** **(SD)**	***P***
stress	13.59(3.96)	17.22(3.75)	<0.001^***^	13.8(3.86)	18.26(3.55)	<0.001^***^	13.86(3.87)	18.43(3.53)	<0.001^***^

ANX, anxiety; DEP, depression; M, median; Q, quartiles; μ, mean; SD, standard deviation.

*p* < 0.05 was considered statistically significant.

* *p* < 0.05,

** *p* < 0.01,

*** *p* < 0.001.

The prevalence of depression was 37.7% (95% CI = 29.7–45.6%). The mean total score of the PHQ-9 was 4.32 (SD = 4.79). The prevalence of anxiety was 26.0% (95% CI = 18.8–33.2%). The mean total GAD-7 score was 2.84 (SD = 4.05). The prevalence of combined depression and anxiety was 24.0% (95% CI = 17.0–31.0%). The mean total ISI score was 4.79 (SD = 4.43). The mean total PSS-10 score was 14.96 (SD = 4.25). Spearman's rank-order correlation analysis revealed that depression and anxiety had a significant correlation (correlation coefficient = 0.73, *P* < 0.01).

### Subgroup analysis

The depression and non-depression groups: The difference significance test revealed that young front-line clinicians with depression were more likely to suffer from severe stress (*P* < 0.01) and insomnia (*P* < 0.01) in high-risk areas during the COVID-19 sporadic epidemic. The prevalence of depression varied significantly across education levels (*P* < 0.05) and inhabitation (*P* < 0.01). In addition, responses to the questions about attitudes toward the long-term side effects of the COVID-19 vaccines were significantly different between the depression and non-depression groups (*P* < 0.05) (Table 1).

The anxiety and non-anxiety groups: The difference significance test revealed that young front-line clinicians with anxiety were more likely to have more severe stress (*P* < 0.01) and insomnia (*P* < 0.01) in high-risk areas during the COVID-19 sporadic epidemic. The prevalence of anxiety was significantly different by inhabitation (*P* < 0.05). Responses to the questions about attitudes toward COVID-19 vaccines except for the protective effects of COVID-19 vaccines were significantly different between the two groups (all *P* < 0.05) (Table 1).

The depression and anxiety comorbid and non-comorbid groups: The difference significance test revealed that young front-line clinicians with anxiety were more likely to suffer from severe stress (*P* < 0.01) and insomnia (*P* < 0.01) in high-risk areas during the COVID-19 sporadic epidemic. The prevalence of anxiety-depression comorbidity was significantly different by inhabitation (*P* < 0.05). In addition, responses to the questions about attitudes toward COVID-19 vaccines except for the protective effects of COVID-19 vaccines were significantly different between the two groups (all *P* < 0.05) (Table 1).

### Factors influencing anxiety, depression, and anxiety-depression comorbidity

Table 2 presents the results of the binary logistic regression analysis. In the multivariate analysis, severe stress (OR = 1.258, 95% CI = 1.098–1.442, *P* < 0.01) and insomnia (OR = 1.282, 95% CI = 1.135–1.447, *P* < 0.01) were positively correlated with depression. Severe stress (OR = 1.487, 95% CI = 1.213–1.823, *P* < 0.01) and insomnia (OR = 1.131, 95% CI = 1.003–1.274, *P* < 0.05) were positively correlated with anxiety. The belief that the vaccine will stop the global epidemic (OR = 0.099, 95% CI = 0.014–0.715, *P* < 0.05) was negatively correlated with anxiety. Severe stress (OR = 1.532, 95% CI = 1.228–1.912, *P* < 0.01) was positively correlated with anxiety-depression comorbidity. Insomnia (OR = 1.081, 95% CI = 0.963–1.214, *P* > 0.05) was not correlated with anxiety-depression comorbidity. The belief that the vaccine will stop the global epidemic (OR = 0.101, 95% CI = 0.014–0.744, *P* < 0.05) was negatively correlated with anxiety-depression comorbidity.

**Table 2:** The binary logistic regression analysis of depression, anxiety, and combined depression and anxiety in the study participants.

**Variable**	**Depression**			**Anxiety**			**Combined depression and anxiety**		
	***P***	**OR**	**95% CI**	***P***	**OR**	**95% CI**	***P***	**OR**	**95% CI**
**Education level (ref: bachelor's degree)**									
Master's degree	0.716	1.228	0.406–3.714	–	–	–	–	–	–
Doctoral degree	0.638	0.588	0.065–5.358	–	–	–	–	–	–
**Inhabitation (ref: alone)**									
With family	0.133	0.320	0.073–1.413	0.941	1.074	0.162–7.114	0.527	0.516	0.067–4.008
Others	0.225	2.012	0.650–6.222	0.128	3.401	0.703–16.447	0.123	3.459	0.716–16.713
**Worried about being infected with COVID-19 (ref: no)**									
Fair	–	–	–	0.382	1.707	0.515–5.659	0.550	1.447	0.432–4.848
Very much	–	–	–	0.182	2.883	0.609–13.655	0.238	2.600	0.531–12.718
**Though vaccines are safe (ref: not safe with obvious side effects)**									
No idea	0.991	1.008	0.256–3.971	0.282	3.496	0.357–34.196	0.418	2.533	0.268–23.958
Safe with no or minimal side effects	0.183	3.272	0.571–18.736	0.334	3.584	0.269–47.676	0.507	2.387	0.183–31.162
**Though vaccines will stop the global epidemic (ref: no)**									
No idea	–	–	–	0.706	0.781	0.216–2.824	0.425	0.591	0.162–2.155
Yes	–	–	–	0.022^*^	0.099	0.014–0.715	0.024^*^	0.101	0.014–0.744
Insomnia	<0.001^***^	1.282	1.135–1.447	0.044^*^	1.131	1.003–1.274	0.187	1.081	0.963–1.214
Stress	0.001^**^	1.258	1.098–1.442	<0.001^***^	1.487	1.213–1.823	<0.001^***^	1.532	1.228–1.912

CI, confidential interval; OR, odds ratio; Ref, reference group.

*p* < 0.05 was considered statistically significant.

* *p* < 0.05,

** *p* < 0.01,

*** *p* < 0.001.

### Factors influencing overall quality of life

Table 3 presents the results of multiple linear regression analysis. In the analysis, severe stress (B = −0.068, 95% CI = −0.129 to −0.007, *P* < 0.05) and insomnia (B = −0.127, 95% CI = −0.188 to −0.067, *P* < 0.01) were negatively correlated with overall QOL. The belief that the vaccine could provide protection (B = 1.442, 95% CI = 0.253–2.631, *P* < 0.05) was positively correlated with overall QOL.

**Table 3:** Multiple linear regression analysis of quality-of-life related factors.

**Variable**	**B**	***P***	**95% CI**	**VIF**
Gender (ref: male)	−0.206	0.383	−0.673 to 0.260	1.204
**Education level (ref: bachelor's degree)**
Master's degree	−0.412	0.148	−0.971 to 0.147	1.541
Doctoral degree	−0.003	0.995	−0.944 to 0.938	1.683
Marriage (ref: single)	−0.811	0.080	−1.719 to 0.098	2.286
Fertility (ref: none)	0.027	0.964	−1.121 to 1.174	1.967
**Inhabitation (ref: alone)**
With family	−0.408	0.261	−1.123 to 0.308	2.346
Others	−0.418	0.179	−1.030 to 0.195	2.169
**Worried about being infected with COVID-19 (ref: no)**
Fair	0.199	0.450	−0.322 to 0.720	1.578
Very much	0.784	0.050	−0.001 to 1.570	1.707
**Thought COVID-19 vaccines could provide protection (ref: no)**
No idea	0.514	0.081	−0.065 to 1.092	1.583
Yes	1.442	0.018^*^	0.253 to 2.631	1.510
**Thought vaccines are safe (ref: not safe with obvious side effects)**
No idea	0.258	0.458	−0.429 to 0.946	2.165
Safe with no or minimal side effects	0.262	0.557	−0.618 to 1.141	2.143
**Thought vaccines will stop the global epidemic (ref: no)**
No idea	0.052	0.876	−0.603 to 0.706	2.374
Yes	0.323	0.415	−0.457 to 1.103	2.085
Depression	−0.047	0.353	−0.146 to 0.052	5.216
Anxiety	−0.062	0.249	−0.168 to 0.044	4.270
Insomnia	−0.127	<0.001^***^	−0.188 to −0.067	1.678
Stress	−0.068	0.029^*^	−0.129 to −0.007	1.573

B, regression coefficient; CI, confidential interval; Ref, reference group; VIF, variance inflation factor.

*p* < 0.05 was considered statistically significant.

* *p* < 0.05,

^**^p < 0.01,

*** *p* < 0.001.

## Discussion

To our knowledge, this is the first survey on the mental health status of young front-line clinicians in high-risk areas during the COVID-19 sporadic epidemic. In this study, we found that the prevalence rates of depression and anxiety among young clinicians were 37.7 and 26.0%, respectively. A study on the psychological status of Chinese adults during the epidemic showed that the prevalence of anxiety and depression in the general population was 7.6 and 11.3%, respectively (3). The different prevalence rates may be related to the higher risk of infection, unpredictable work patterns, and the high psychological stress of clinicians in high-risk areas during the epidemic. In addition, isolation measures lead to the absence of interpersonal communication. If anxiety and depression are more likely to occur, they worsen in the absence of interpersonal communication (45). Another meta-analysis showed that the prevalence of anxiety and depression among clinicians was 21.73 and 25.37%, respectively, during the epidemic (46). The high prevalence of anxiety and depression among young clinicians could be attributed to them having more anxiety characteristics, more difficulty relaxing, and more difficulty adapting to changes than older clinicians (20, 21). Young people show lower levels of wellbeing and optimism than older people, which may also be a risk factor for their vulnerability to anxiety and depression (22).

Our study showed a significant correlation between depression and anxiety (*P* < 0.01). The connection between depression and anxiety is duplex; anxiety can lead to depression, and vice versa (47, 48). This may be related to the decrease in the anterior regions of the default mode network and the increased connectivity in the posterior regions (49). Previous studies have shown that anxiety-depression comorbidity was highly prevalent during the SARS pandemic (50). In this study, the comorbidity rate of depression and anxiety disorder was 23.97%. Because of the similar pathogenesis underlying depression and anxiety (51), we speculate that anxiety-depression comorbidity may be the result of the COVID-19 sporadic epidemic in terms of mental illness.

Insomnia is more severe in individuals with depression or anxiety. According to relevant studies, insomnia can damage emotional regulation and increase the risk of depression or anxiety (52–54). However, the relationship between insomnia and depression or anxiety may be bidirectional (52). Many studies point out that depression or anxiety can reduce the quality of sleep, leading to insomnia (17, 55, 56). Serotonergic and dopaminergic dysfunctions may be the common underlying mechanism of insomnia and mental disorders (57). In addition, depression, anxiety, and insomnia may also have a common genetic basis (58). Interestingly, no correlation was found between anxiety-depression comorbidity and insomnia in this study. This is different from the results of previous studies (47, 58). The differences may be due to the use of different survey tools or different study populations. However, this was only a preliminary result that needs further confirmation from additional studies. Faced with the sporadic epidemic, the working hours and labor intensity of clinicians in high-risk areas have increased, leading to insufficient rest time and psychological distress. In conclusion, COVID-19 plays an important role in triggering or aggravating mental health conditions such as depression, anxiety, and insomnia.

Our study showed that stress is a risk factor for anxiety and/or depression. This is consistent with the findings of previous studies (59). Therefore, to promote the mental health of clinicians, it is necessary to develop personalized intervention measures to reduce stress during the COVID-19 sporadic epidemic.

Our study showed that QOL was determined by the interaction between protective factors (e.g., the belief that the vaccine could provide protection) and risk factors (e.g., severe insomnia and stress conditions). Adequate sleep and reasonable stress relief are considered indispensable elements of health, general wellbeing, and proper daily functioning. Stress and insomnia might reduce clinicians' QOL by leading to cognitive dysfunction (60), physical discomfort (61), and job burnout (62). Further studies on the sleep patterns and stress management strategies of young front-line clinicians in high-risk areas are needed to develop strategies to prevent or alleviate problems and improve the QOL.

Currently, the absence of proven treatments for COVID-19 has led the world's population to pin their hopes for vaccines (63). After the outbreak of the epidemic, the Chinese government urgently developed a vaccine, and the Chinese population reflected the strong demand and high acceptance of the importance of COVID-19 vaccines (64). A global survey of potential acceptance of the COVID-19 vaccine showed that Chinese people's acceptance of the vaccine was nearly 90% (64). Our study showed that young front-line clinicians in high-risk areas who thought that the vaccine could stop the global epidemic were less prone to anxiety and anxiety-depression comorbidity. Raising confidence in and awareness of vaccines may help address the mental health problems of young front-line clinicians in high-risk areas. Although our sample comprised young front-line clinicians, not all clinicians work in infectious diseases departments, and some have relatively poor knowledge of vaccines. The dissemination of misinformation could have a significant impact on confidence in the COVID-19 vaccine, further exacerbating mental health problems among the young front-line clinician population (65, 66). Therefore, national and local regulatory authorities need to conduct health education and outreach through authoritative sources to carefully explain the effectiveness of the vaccine, the duration of the antibody, and the importance of achieving group immunity. This increases confidence that the COVID-19 vaccine will end the global epidemic, reduce the prevalence of anxiety and/or anxiety-depression comorbidity, and effectively alleviate specific concerns or misconceptions in high-risk areas.

## Limitation

Our study has several limitations. First, due to the cross-sectional study design, it was difficult to make a causal inference. Second, the sample size of this study was limited, and single-area studies may have limited applicability and generalizability to clinicians in other high-risk areas. Third, due to the sudden occurrence of the COVID-19 disaster, we were unable to assess the psychological status of the respondents before the sporadic epidemic. Fourth, depression, anxiety levels, and other related factors, such as sleep disturbance and stress levels, were measured by self-report questionnaires, without objective indicators of related factors in this study. Finally, social support plays a pivotal role in reducing the likelihood of psychological impact and QOL (67), but it was not evaluated in this study.

## Conclusion

We identified the main mental health problems of young front-line clinicians in high-risk areas during the COVID-19 sporadic epidemic in China. Depression, anxiety, anxiety-depression comorbidity, and QOL were associated with many factors, including insomnia, stress, and a portion of attitudes toward the COVID-19 vaccine. Due to the reasonable epidemic prevention and control measures and popularization of vaccination taken by the Chinese government, there has been no recent large-scale outbreak of the epidemic in China. The sporadic epidemic may become the most important problem for the prevention and control of the epidemic in the future. Therefore, establishing early targeted mental health interventions for young clinicians in high-risk areas during the COVID-19 sporadic epidemic situation should be part of global preparedness efforts.

## Data availability statement

The raw data supporting the conclusions of this article will be made available by the authors, without undue reservation.

## Author contributions

XS and YZha contributed to the study design, analyzed the data, and wrote this manuscript. QL and YZhu did the online survey, data collection, and logical check. GZ, YL, and JZ revised the manuscript. All authors reviewed and approved the manuscript.

## Funding

This work was funded by Natural Science Foundation of Shandong Province (ZR2017 LH036) and Key R & D Projects of Jining City: Psychological Rescue Mode for COVID-19 Pneumonia (2021YXNS078).

## Conflict of interest

The authors declare that the research was conducted in the absence of any commercial or financial relationships that could be construed as a potential conflict of interest.

## Publisher's note

All claims expressed in this article are solely those of the authors and do not necessarily represent those of their affiliated organizations, or those of the publisher, the editors and the reviewers. Any product that may be evaluated in this article, or claim that may be made by its manufacturer, is not guaranteed or endorsed by the publisher.
